# Leukemoid reaction in a patient with adenocarcinoma of the lung: a case report

**DOI:** 10.1186/1752-1947-6-211

**Published:** 2012-07-19

**Authors:** Hendrik Riesenberg, Frauke Müller, Martin Görner

**Affiliations:** 1 Department of Oncology, Hematology and Bone Marrow Transplantation with Section of Pneumology, University Medical Center Hamburg-Eppendorf, Martinistraße 52, 20246 Hamburg, Germany; 2 Department of Hematology and Oncology, Community Hospital Bielefeld, Teutoburger Strasse 50, 33604 Bielefeld, Germany

## Abstract

**Introduction:**

Lung cancers are characterized by high incidence, prevalence and mortality. They may be associated with numerous paraneoplastic syndromes. Mild leukocytosis is not rare. The case described here, however, is of a female patient with adenocarcinoma of the lung who developed extreme leukocytosis at over 140,000 cells/μL. Descriptions of such leukemic forms of lung cancer are few and far between in the literature. In our case, the complete hematological diagnostic investigation, which included cytological, immunocytological, cytogenetic, histological and molecular genetic tests of the bone marrow (mutation analyses of *BCR-ABL* and *JAK2*), was accompanied for the first time by a molecular genetic workup of the primary tumor for *epidermal growth factor receptor* and *K-RAS* gene mutations.

**Case presentation:**

We present the medical case of a 51-year-old female Caucasian patient, who was diagnosed with a poorly differentiated stage IV (International Union Against Cancer staging) adenocarcinoma of the lung. While undergoing treatment, our patient developed extreme leukocytosis, for which, despite extensive diagnostic tests, no infection-related or hematological cause could be identified. The tumor proved to be highly resistant to treatment. Our patient died only five months after the initial diagnosis.

**Conclusion:**

A leukemoid course can most likely be interpreted as the paraneoplastic production of hematopoietic growth factors. Despite the absence of a verified primary hematological origin, this possibility should always be investigated in all patients in a comparable situation.

## Introduction

Both in Europe and in the US, lung cancer is by far the most common fatal cancer in both sexes [1,2]. The classic symptoms include coughing, dyspnea and hemoptysis. Particularly in small cell but also in non-small cell lung cancer, paraneoplastic syndromes are observed that, in terms of their characteristics and clinical relevance, should be classified very differently. The production of hormones or hormone precursors could result in endocrine paraneoplasia. Examples include the tumor-related production of parathyroid hormone, adrenocorticotropic hormone, antidiuretic hormone, thyroid-stimulating hormone, insulin, erythropoietin, calcitonin and serotonin. These are distinct from antibody-mediated paraneoplasia, which may result in unusual skin changes (for example, dermatomyositis) but also in severe neurological disorders (for example, myasthenia, Lambert-Eaton syndrome).

It is not unusual for both small cell and non-small cell lung cancers to be associated with mild leukocytosis. These should be distinguished from the leukemoid courses of non-small cell lung cancer, especially large cell carcinoma, occasionally described in the literature, where the development of extreme leukocytosis at over 50,000 cells/μL has been observed.

In the following case description, the origin of the leukemoid course is discussed in detail under both infection-related and hematological aspects, and explanations offered for the resistance to treatment. In addition, molecular genetic investigations of the primary tumor are presented for the first time.

## Case presentation

We report on a 51-year-old female Caucasian patient with no significant previous illnesses. Our patient had been a smoker for many years, with persistent nicotine use at the time of her first presentation. The initial symptom, which first led the patient to contact her primary care physician, was pain in the region of her left hip. She did not complain of cough, dyspnea, fever or night sweats. Her weight remained constant.

A computed tomography (CT) scan of her left hip joint demonstrated extensive osteolysis in the acetabulum with infiltration of the iliopsoas muscle. Moreover, bone fracture of the joint socket was highly suspected. Magnetic resonance tomography (MRT) of her pelvis confirmed the pathological fracture. Skeletal scintigraphy found no further evidence of osteolysis.

We performed a CT-guided biopsy of the large area of osteolysis in her left acetabulum. The histological diagnosis was of a poorly differentiated adenocarcinoma. Despite extensive immunohistological chemical analyses (pan-keratin focally positive; cytokeratin 8/18 moderately positive; cytokeratin 5/6 weakly positive; cytokeratin 7 moderately to strongly positive; melanoma cocktail, thyroid transcription factor-1, mammaglobin, thyroglobulin, cluster of differentiation (CD) 56 and CD 138 negative), it proved impossible to assign the primary tumor to a particular organ.

Moderate leukocytosis at 21,200 cells/μL was diagnosed as early as this point, in the presence of mild anemia and normal platelet concentration. No leukocyte differentiation was performed at this time. A routine blood count from one and a half years before, examined retrospectively for this analysis, was without pathological findings.

**Figure 1:**
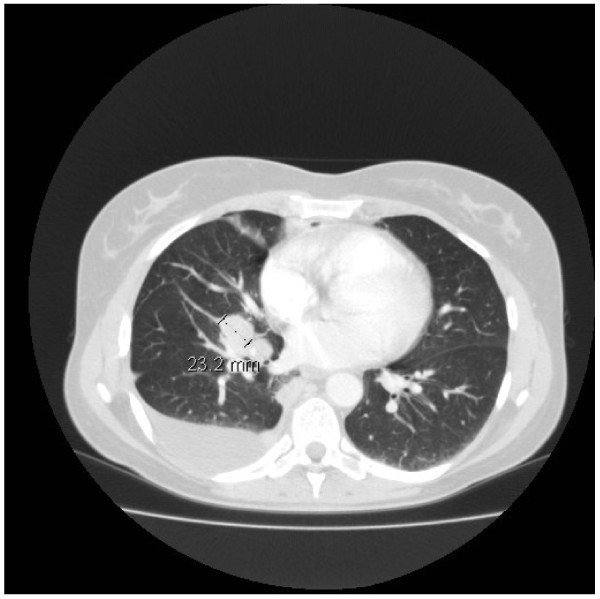
** Primary lesion.** Pulmonary tumor in the right hilar region with a diameter of 2.3cm in close proximity to the upper lobe and encircling the right main bronchus.

A CT scan of her neck, chest and abdomen and a MRT scan of her cranium were performed. These demonstrated a pulmonary tumor in her right hilar region with a diameter of 2.3cm, in close proximity to the upper lobe and encircling her right main bronchus (Figure 1). Enlarged lymph nodes up to 2.5cm in size and infracarinal lymph nodes up to 2.0cm in size were observed in her right hilar region. In addition, numerous pleural, pericardial and diaphragm metastases were diagnosed. No pulmonary metastases were seen. Excess tissue was shown in her right infraclavicular region. Abdominally, a distension of her left adrenal of 2.0cm in diameter was assessed as highly suspect for metastasis. The cranial MRT findings were normal.

The leukocyte concentration in the mean time was elevated at 39,000 cells/μL, with largely unremarkable differentiation (see Table 1).

**Table 1:** Blood count and infection parameters

**Serum concentration**	**29 Apr 2008**	**11 Nov 2010**	**25 Nov 2010**	**14 Feb 2011**
Leukocytes (cells/μL)	7900	21,200	39,000	146,200
Hemoglobin (g/dL)	15.3	12.8	12.2	8.7
Platelets (cells/μL)	249,000	337,000	372,000	266,000
Neutrophils (%)	-	-	92	85
Lymphocytes (%)	-	-	4	1
Monocytes (%)	-	-	3	2
Eosinophils (%)	-	-	0	0
Basophils (%)	-	-	1	1
Metamyelcytes (%)	-	-	-	6
Myelocytes (%)	-	-	-	5
C-reactive protein (mg/L)	2.8	19.5	43.2	49.3
Procalcitonin	-	-	-	0.4

Bronchoscopy and transbronchial needle aspiration from her main and upper lobe carina demonstrated a moderately to poorly differentiated adenocarcinoma of the lung, consistent with the primary tumor of the known metastasis in her left acetabulum. In molecular pathological follow-up investigations, based on the metastasis of the acetabulum, no mutation of the *epidermal growth factor receptor* gene could be identified (wild type). The *K-RAS* gene exhibited a mutation, which argues in principle against the use of tyrosine kinase inhibitors of the gefitinib type.

The next step involved radiotherapy of the area of osteolysis in her left hip joint, including the surrounding soft tissue metastasis, of up to 40Gy with a single dose of 2Gy. In addition, simultaneous radio- and chemotherapy was started consisting of radiotherapy of the primary tumor and of the mediastinum up to 55.8Gy with a single dose of 1.8Gy plus cisplatin (20mg/m², days 1 to 4 and 29 to 32) and vinorelbine (12.5mg/m², days 1, 8, 15, 29, 36 and 43) was started.

**Figure 2:**
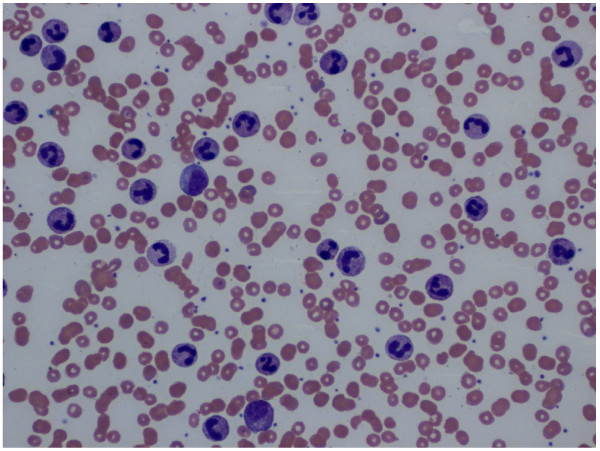
**Peripheral blood smear.** Extreme leukocytosis with largely normal differentiation and complete maturation (neutrophils 85%, lymphocytes 1%, monocytes 2%, eosinophils 0%, basophils 1%, metamyelocytes 6%, myelocytes 5%).

In the course of the therapy, our patient reported progressive pain in the region of her left flank, which was attributable to a considerable increase in the size of the adrenal metastasis to 5.9 × 4.4 × 7.5cm. A blood count at this point showed leukocytosis at 146,200 cells/μL, with largely normal differentiation and complete maturation (Figure 2).

**Figure 3:**
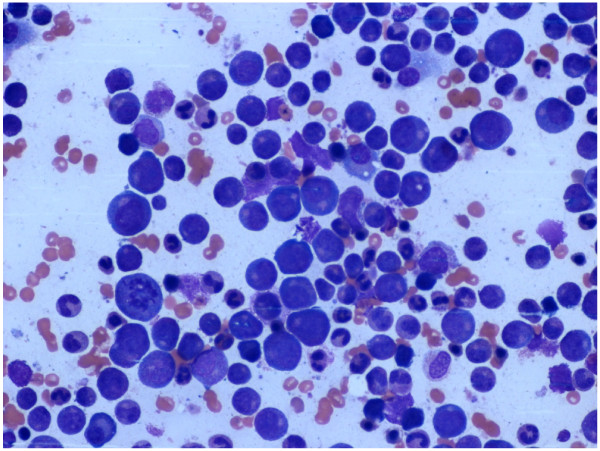
**Bone marrow cytology.** Massively increased and left-shifted granulopoiesis up to the myeloblasts with displaced erythropoiesis and normal megakaryopoiesis, as can typically develop on stimulation with granulocyte colony-stimulating factor (myeloblasts 4%, promyelocytes 12%, myelocytes 15%, metamyelocytes 7%, band cells 13%, segmented neutrophils 31%, proerythroblasts 2%, erythroblasts 16%, lymphocytes 2%).

Inflammation parameters were, at most, mildly elevated (C-reactive protein 49.3mg/L, procalcitonin. 0.4). She had no fever, so we assumed not so much an inflammatory reaction or abscess formation but rather feared a hematological secondary disorder. Her levels of liver transaminases were normal (aspartate transaminase 17U/L, alanine transaminase 21U/L). A cytological bone marrow examination showed a massively increased and left-shifted granulopoiesis up to the myeloblasts with displaced erythropoiesis and normal megakaryopoiesis, as can typically develop under stimulation with granulocyte colony-stimulating factor (G-CSF) (Figure 3). In a fluorescence activated cell sorting analysis, granulocytic maturation of cells with partial coexpression of CD56 was predominantly seen. No immature cells were detected; in principle, these findings seemed consistent with the presence of myeloproliferative neoplasia.

Cytogenetic investigation showed a normal female chromosome set (46,XX). Molecular genetic analysis by means of fluorescence *in situ* hybridization ruled out t(9;22) translocation. As expected, polymerase chain reaction also failed to demonstrate a *BCR-ABL* fusion gene. Similarly, a V617F mutation in the *JAK2* gene could also not be demonstrated, which overall makes the presence of a myeloproliferative neoplasia very unlikely although it does not rule it out. On histology, a bone marrow biopsy also provided no evidence supporting either a hematological neoplasia, in particular chronic myelogenous leukemia. (CML), or the presence of infiltration by tumor cells.

Although our patient continued to exhibit no classic signs of inflammation, the differential diagnosis of abscess formation in the soft tissue metastasis in the iliopsoas muscle had to be followed up, given the absence of evidence for a hematological secondary disorder. However, this could not be confirmed by means of CT-guided biopsy. The biopsy specimen proved to be just as sterile as the repeatedly taken blood and urine cultures.

At the same time, the empirically initiated antibiotic treatment, first with ceftriaxone and subsequently with piperacillin and sulbactam sodium and also metronidazole, failed to affect either the leukocyte concentration or the C-reactive protein and procalcitonin to a clinically relevant degree.

Upon completion of the simultaneous radio- and chemotherapy, the primary tumor exhibited a slight regression. The adrenal metastasis, however, had increased in size during the ongoing therapy to 8.0 × 4.8cm. We therefore attributed the regression of the primary tumor first and foremost to the radiotherapy and initiated systemic second-line therapy with docetaxel (30mg/m², once a week) and pemetrexed (500mg/m², repeated on day 22). As there was no clinical response and our patient’s general condition deteriorated continuously, this therapy was discontinued after just one cycle. A third-line therapy with gemcitabine (1,000mg/m², once a week) failed to have an effect either on the primary tumor or on the leukocyte concentration. Our patient died two weeks after starting the third-line therapy.

## Discussion

Leukemoid courses of various tumor entities, especially gastrointestinal, urogenital, head-neck and lung cancers, have been described in the literature [3]. The development can be affected by a variety of factors. On the one hand, this may involve the presence of an infection or abscess formation. Equally, leukocytosis may be induced in the short term by the administration of high-dose corticosteroids. Another possible cause that needs to be considered is an existing or secondary hematological neoplasia, which develops following previous therapy [4,5]. The cause most discussed in the literature, however, is paraneoplastic production of hematopoietic growth factors. Asano *et al.* published the first report of CSF-producing lung cancers, characterized by the development of extreme neutrophilia. The neutrophilia was transferred to nude mice by the transplantation of tumor cells [6]. Likewise, several subsequent investigations demonstrated elevated serum concentrations of the hematopoietic growth factors G-CSF, granulocyte-monocyte (GM)-CSF and also interleukin-6 in patients with lung cancer and extreme neutrophilia [7-9].

In an investigation published by Kasuga *et al*., out of 33 patients with tumor-associated leukocytosis (leukocytes from 11,400 cells/μL to 190,000 cells/μL) and elevated serum concentration of various hematopoietic growth factors demonstrated by means of enzyme-linked immunoassay, only one patient exhibited small cell lung cancer. All the other patients exhibited non-small cell lung cancers, with the highest incidence being that of large cell carcinoma. In the case of the severe leukemoid courses (leukocytes >50,000 cells/μL), patients with large cell carcinoma also showed the highest incidence at 27.8%, whereas leukocyte concentrations above 50,000 cells/μL were demonstrated in just 0.8% of the identified adenocarcinomas and in 3.3% of the squamous epithelial carcinomas [10].

In a single case report on a patient with a leukemic course of a lung cancer, differentiation from CML was provided by a bone marrow biopsy; but no conclusions were reached on the precise histology of the primary tumor or with regard to molecular genetics [11].

As an example, we may refer here to the study by Shalom *et al*., which reports two patients (a 61-year-old woman with poorly differentiated large cell carcinoma and a 43-year-old man with poorly differentiated squamous epithelial carcinoma) in an extensively metastasized situation at the initial diagnosis, who, during the course of the illness, exhibited leukocyte concentrations exceeding 150,000 cells/μL with the neutrophil granulocyte fraction exceeding 90%. In both cases, neither the infection-related parameters nor the polymerase chain reaction for *BCR-ABL* proved conclusive for ruling out CML. No further hematological investigations or analysis of the primary tumor were performed. Both patients failed to benefit at any time from their chemotherapy, and died only four and seven months after the initial diagnosis [12].

In the case described here, extensive diagnostic tests ruled out infection-related and primary hematological causes for the leukocytosis. A paraneoplastic origin seems likely. It is known that, to a high degree, epithelial tumors can express various forms of the G-CSF receptor and that cell proliferation may occur after ligand binding [13]. Paraneoplastic production of growth factors by the tumor itself would mean permanent stimulation of these tumor cells and explain the high aggressiveness and the uncontrollable progression. It is impossible to judge at this time if potential G-CSF and GM-CSF-stimulated signal transduction can be influenced by medication.

## Conclusions

In rare cases, lung cancer can exhibit a leukemoid course. Where there is autonomous production of hematopoietic growth factors by the tumor cells themselves and concurrent, uncontrollable proliferation, the prognosis for such lung cancers is very poor. To distinguish between this paraneoplastic phenomenon and primary hematological or therapy-related secondary neoplasia, extensive hematological diagnostic tests should always be carried out.

## Consent

Written informed consent was obtained from the patient’s next of kin for publication of this case report and accompanying images. A copy of the written consent is available for review by the Editor-in-Chief of this journal.

## Competing interests

The authors declare that they have no competing interests.

## Authors’ contributions

HR wrote the case report. HR and FM analyzed and interpreted the patient data relating to the oncological disease. MG analyzed and interpreted the patient data relating to the oncological disease and contributed to the writing and revision of the manuscript. All authors read and approved the final manuscript.
